# Temporal muscle thickness and area with various characteristics data of the patients with aneurysmal subarachnoid hemorrhage who underwent endovascular coiling

**DOI:** 10.1016/j.dib.2020.105715

**Published:** 2020-05-18

**Authors:** Masahito Katsuki, Yasuhiro Suzuki, Keiko Kunitoki, Yoshimichi Sato, Keisuke Sasaki, Shoji Mashiyama, Ryo Matsuoka, Elissa Allen, Hibiki Saimaru, Ryota Sugawara, Akinori Hotta, Teiji Tominaga

**Affiliations:** a Department of Neurosurgery, Iwaki City Medical Center, 16 Kuzehara, Mimayamachi, Uchigo, Iwaki, Fukushima 973-8555, Japan; b Harvard School of Public Health, 677 Huntington Ave, Boston, MA, USA; c Resident, Iwaki City Medical Center, 16 Kuzehara, Mimayamachi, Uchigo, Iwaki, Fukushima 973-8555, Japan; d Department of Neurosurgery, Tohoku University Graduate School of Medicine, 1-1 Seiryo, Aobaku, Sendai, Miyagi, 980-8574, Japan

**Keywords:** Cerebral aneurysm, Coiling, Prognostic factor, Sarcopenia, Skeletal muscle, Subarachnoid hemorrhage, Temporal muscle

## Abstract

These data present the characteristics of patients with subarachnoid hemorrhage who underwent endovascular coiling. We retrospectively collected data from the medical records of Iwaki City Medical Center including physiological symptoms, laboratory data, radiological data on admission, and modified Rankin Scale scores at 6 months. Our article entitled “Temporal Muscle as an Indicator of Sarcopenia is Independently Associated with Hunt and Kosnik Grade on Admission and the Modified Rankin Scale at 6 Month of Patients with Subarachnoid Hemorrhage Treated by Endovascular Coiling” was based on these data [1]. We previously reported similar small dataset of elderly patients with subarachnoid hemorrhage who underwent surgical clipping [2], [3]. However, remarkably, this is the largest and the first dataset on temporal muscle thickness or area of patients of all ages with subarachnoid hemorrhage who underwent endovascular coiling, not surgical clipping.

Specifications Table

**Table:** 

Subject	Clinical Neurology
Specific subject area	Neurosurgery and Gerontology
Type of data	Table
How data were acquired	We investigated the medical records of Iwaki City Medical Center, and collected data, such as patients’ sex, age, comorbidities, prognosis, laboratory data, radiological data, and neurological and physiological symptoms. We also analyzed computed tomography images to obtain information on temporal muscle thickness and area.
Data format	Raw, and partially averaged.
Parameters for data collection	From the medical records, we collected objective data, such as laboratory data, physiological data, and neurological symptoms. Radiological information, such as temporal muscle thickness and area, could be less objective, so the averages of the left and right areas were calculated from three determinations on each side by two investigators. The intraclass correlation coefficients (2, 2) of temporal muscle thickness and area were 0.882 and 0.786, respectively.
Description of data collection	From the subarachnoid hemorrhage databases of Iwaki City Medical Center, we retrospectively retrieved the data from all 298 patients with aneurysmal subarachnoid hemorrhage who underwent endovascular coiling. We collected data regarding neurological and physiological symptoms and laboratory data on admission from the medical records. Radiological information, including temporal muscle profile, was acquired using computed tomography images. The postoperative clinical course was also investigated. Outcomes were assessed by the modified Rankin Scale at 6 months.
Data source location	Institution: Iwaki City Medical Center City/Town/Region: Iwaki, Fukushima Country: Japan Latitude and longitude (and GPS coordinates) for collected samples/data:] 37.042548532944, 140.86975350334
Data accessibility	With the article
Related research article	Author's name: Masahito Katsuki, Yasuhiro Suzuki, Keiko Kunitoki, Yoshimichi Sato, Keisuke Sasaki, Shoji Mashiyama, Ryo Matsuoka, Elissa Allen, Hibiki Saimaru, Ryota Sugawara, Akinori Hotta, Teiji Tominaga. Title: Temporal Muscle as an Indicator of Sarcopenia is Independently Associated with Hunt and Kosnik Grade on Admission and the Modified Rankin Scale at 6 Month of Patients with Subarachnoid Hemorrhage Treated by Endovascular Coiling. Journal: World Neurosurgery Status: In press. https://doi.org/10.1016/j.wneu.2020.02.033

## Value of the Data

• Large data including temporal muscle bulk and outcomes of the patients with subarachnoid hemorrhage who underwent endovascular coiling.
• This data can be used as a reference value for neurosurgeons when they treat their patients with subarachnoid hemorrhage.
• This data also can be used for further analysis using artificial intelligence to establish the prediction model.
• The data of temporal muscle thickness/area of the patients with subarachnoid hemorrhage can be used to compare healthy patients to investigate the meaning of temporal muscle, focusing on nutrition and sarcopenia.

## 1 Data

The dataset in the table describes the characteristics of patients of all ages with subarachnoid hemorrhage who underwent endovascular coiling. We retrospectively collected data from medical records of Iwaki City Medical Center including neurological and physiological symptoms, laboratory data, radiological data on admission, and modified Rankin Score at 6 months. The detailed data are available in the supplementary file.

**Table:** **Title of the table:** Characteristics of patients with subarachnoid hemorrhage who underwent endovascular coiling in Iwaki City Medical Center from 2009 to 2019.

Variables (n = 298)	Mean±SD or median (range)†
Sex, female:male	208:90
Age	63.7±13.7
Hunt and Kosnik grade	2.62±0.81
Location of the aneurysm, posterior circulation: anterior circulation	276:22
Aneurysm size (mm)	6.05±3.23
Fisher computed tomography scale	3.16±0.64
History of smoking (n = 271)	95 (35%)
History of heavy drinking (n = 271)	63 (23%)
History of hypertension	163 (55%)
History of diabetes mellitus	27 (9%)
History of dyslipidemia	48 (16%)
Intake of antithrombotic drug	42 (14%)
Symptomatic vasospasm	57 (19%)
Ventriculoperitoneal shunt	49 (16%)
Postoperative complications except for symptomatic vasospasm or hydrocephalus	93 (31%)
Modified Rankin Scale at 4 weeks	2.05±2.05
Modified Rankin Scale at 6 months	1.89±2.07
Temporal muscle thickness (mm)	5.64±2.15
Temporal muscle area (mm^2^)	253.7±132.5
Height (cm) (n = 290)	156.8±9.53
Weight (kg) (n = 290)	55.8±12.3
Albumin (mg/dL) (n = 260)	4.13±0.61
White blood cell count (× 10^2^/µL) (n = 290)	107.7±38.3
Total cholesterol (mg/dL) (n = 189)	184.0±42.0
Triglycerides (mg/dL) (n = 244)	114.3±84.6
Low density lipoprotein cholesterol (mg/dL) (n = 205)	112.7±35.0
Blood glucose (mg/dL) (n = 282)	150.9±44.9
HbA1c (%) (n = 206)	5.75±0.73
Potassium (mEq/L) (n = 289)	3.7 (2.2-5.0)

† The Shapiro-Wisk test was used to evaluate the distribution normality. The factors with normal distribution are shown by mean±standard deviation, and those with non-normal distribution are shown by median (range). The detailed data are available in the supplementary file.

## 2 Experimental Design, Materials, and Methods

From our medical records, we retrospectively retrieved the data of 298 patients of all ages with aneurysmal subarachnoid hemorrhage who had been admitted from 2009 to 2019 and treated with endovascular coiling. All patients with these data had been independent in activities in daily living before the onset of subarachnoid hemorrhage. We collected data regarding neurological and physiological symptoms, clinical course, and laboratory data on admission. Neurological severity was assessed using the Hunt and Kosnik grade [4].

We also determined the size of the aneurysm, location of the aneurysm, temporal muscle thickness, and temporal muscle area based on the data of computed tomography and computed tomography angiography on admission. We used Aquilion Prime SP (Canon Medical Systems Corporation, Tochigi, Japan) to obtain computed tomography and computed tomography angiography images of 0.5 × 0.5 × 1.0 mm voxels. The slice thickness was reconstructed to 5 mm. The window width was adjusted to 300, and the window level was adjusted to 20. The temporal muscle thickness and area were measured by two investigators who did not know the patients’ outcomes using SYNAPSE version 4.1.5 imaging software (Fujifilm Medical, Tokyo, Japan). The temporal muscle thickness was measured bilaterally perpendicular to the long axis of the temporal muscle at the slice 5 mm above the orbital roof and calculated using averages of the left and right areas from three determinations of each side. The temporal muscle area was measured manually by tracing the outline of the temporal muscle on the same slice as that used for measuring the temporal muscle thickness and computed using the software. The averages of the left and right areas from three determinations of each side were calculated by two investigators [1], [2], [3].

Symptomatic vasospasm was diagnosed by computed tomography angiography, magnetic resonance imaging, or magnetic resonance angiography with symptoms. Computed tomography was used to diagnose hydrocephalus with symptoms. Outcomes were assessed using the modified Rankin Scale [5] at 4 weeks and 6 months.

## Declaration of Competing Interest

The authors declare that they have no known competing for financial interests or personal relationships which have, or could be perceived to have, influenced the work reported in this article.

## Appendix Supplementary materials

Image, application 1
